# Influence of Gadoxetate disodium to the hepatic proton density fat fraction quantified with the Dixon sequences in a rabbit model

**DOI:** 10.1007/s00261-024-04320-w

**Published:** 2024-04-29

**Authors:** Xia Wang, Sheng Zhang, Zhe Huang, Gang Tian, Xiaofan Liu, Lijun Chen, Liang An, Xumiao Li, Ningna Liu, Yang Ji, Yuedong Han

**Affiliations:** 1 Department of Radiology, Xi’an GaoXin Hospital, Xi’an Jiao Tong University, South Tuanjie Rd 16, Xi’an, 710075 Shaanxi China; 2 Department of Clinical Laboratory, Xi’an GaoXin Hospital, Xi’an, China; 3 Department of Pathology, Xi’an GaoXin Hospital, Xi’an, China; 4 Department of Imaging Center, First Affiliated Hospital, Xi’an Medical University, Shaanxi, China

**Keywords:** Rabbit, Metabolic dysfunction-associated steatotic disease (MASLD), Gadoxetate disodium (Gx), VIBE Dixon, Proton density fat fraction (PDFF)

## Abstract

**Objective:**

To study the impact of Gx on quantification of hepatic fat contents under metabolic dysfunction-associated steatotic liver disease (MASLD) imaged on VIBE Dixon in hepatobiliary specific phase.

**Methods:**

Forty-two rabbits were randomly divided into control group (*n* = 10) and high-fat diet group (*n* = 32). Imaging was performed before enhancement (Pre-Gx) and at the 13th (Post-Gx13) and 17th (Post-Gx17) min after Gx enhancement with 2E- and 6E-VIBE Dixon to determine hepatic proton density fat fractions (PDFF). PDFFs were compared with vacuole percentage (VP) measured under histopathology.

**Results:**

33 animals were evaluated and including control group (*n* = 11) and MASLD group (*n* = 22). Pre-Gx, Post-Gx13, Post-Gx17 PDFFs under 6E-VIBE Dixon had strong correlations with VPs (*r*^2^ = 0.8208—0.8536). PDFFs under 2E-VIBE Dixon were reduced significantly (*P* < 0.001) after enhancement (*r*^2^ = 0.7991/0.8014) compared with that before enhancement (*r*^2^ = 0.7643). There was no significant difference between PDFFs of Post-Gx13 and Post-Gx17 (*P* = 0.123) for which the highest consistency being found with 6E-VIBE Dixon before enhancement (*r*^2^ = 0.8536). The signal intensity of the precontrast compared with the postcontrast, water image under 2E-VIBE Dixon increased significantly (*P* < 0.001), fat image showed no significant difference (*P* = 0.754).

**Conclusion:**

2E- and 6E-VIBE Dixon can obtain accurate PDFFs in the hepatobiliary specific phase from 13 to 17th min after Gx enhancement. On 2E-VIBE Dixon (FA = 10°), effective minimization of T1 Bias by the Gx administration markedly improved the accuracy of the hepatic PDFF quantification.

**Graphical abstract:**

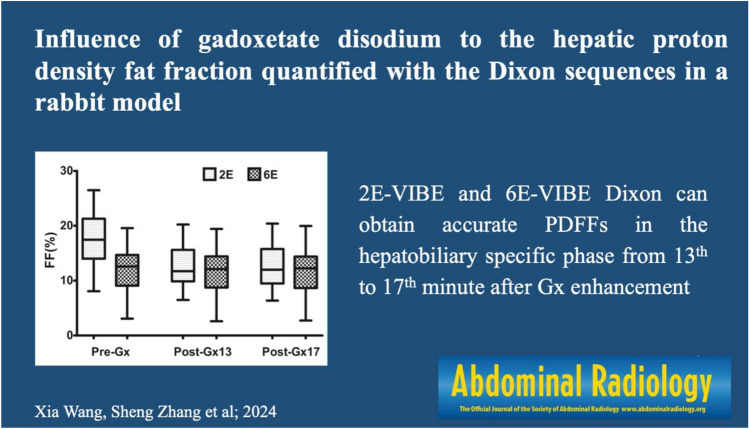

## Introduction

Metabolic dysfunction-associated steatotic disease (MASLD), which used to be the name of non-alcoholic fatty liver disease (NAFLD), refers to the excessive accumulation of triglycerides within the hepatocytes [1, 2]. MASLD is reversible with timely diagnosis and treatment [1]. Otherwise, it can develop from non-alcoholic steatohepatitis, liver fibrosis, cirrhosis and even to liver cancer [1–3]. Liver biopsy is the gold standard for the diagnosis and staging of MASLD, but it is difficult to be used for health screening or longitudinal monitoring of treatment effects due to its invasive procedure [3, 4].

Magnetic resonance imaging can be used to observe liver fat content noninvasively [5]. Magnetic resonance spectroscopy (MRS) can obtain the hepatic proton density fat fraction (PDFF) based on the peak value of proton resonance frequency [6]. Hepatic PDFF measured by using MRS (hereafter, MRS-PDFF) has high accuracy and repeatability, and it can be considered the gold standard of noninvasive liver fatty measurement. However, it has high requirements for equipment, long acquisition time and complex postprocessing program, thus obviating its being widely used [7, 8]. Fat–water separation method with in-phase and out-of-phase was used to evaluate the feasibility of PDFF quantification, but its clinical application was restricted to the abilities of only obtaining the qualitative imaging [8, 9]. Thereafter, different variants of this technique, 2-echoes, 3-echoes, 6-echoes, and even 15-echoes, were developed and applied in the clinical trails for acquiring more accurate PDFF [6, 7]. The most commonly used techniques are the 2-echoes and 6-echoes in the commercial equipment. Dixon technique can complete hepatic PDFF examination with a single breath-hold, for which the errors caused by T1 bias and T2* attenuation can be overcome to a great extent [10–12]. Meta-analysis shows an excellent linear relationship between the results of hepatic PDFF measured by using Dixon technique (hereafter, Dixon-PDFF) and MRS-PDFF, which can be used as an accurate and noninvasive method to evaluate fat contents of the liver [13].

Gadoxetate disodium (Gx) can be used as an on specific contrast agent for conventional dynamic three-phase scanning of the liver which can be finished in 10 min. Furthermore, Gx can be taken up specifically by hepatocytes after intravenous injection, with its peak concentration in the liver at about 20–40 min, during which time hepatobiliary specific MR scans are often performed clinically [14–16]. On the other hand, Gx can be secreted from hepatocytes into the bile duct system at 5 min and the gallbladder 13 min after intravenous injection respectively [14–16]. How to take advantage of the long time interval from 10 to 20 min between the conventional dynamic liver MR scans and the hepatobiliary specific MR scan is an important problem for efficient utilization of Gx enhanced MR scanning. Dixon-PDFF measurements have been performed about 20 min after Gx (0.05 mmol/kg) enhancement and found that it can be accurately obtained at this time point [17, 18]. However, whether it could be accurately measured in the time interval before the time point above should be further investigated. Herein, the authors established a rabbit model of MASLD and scanned it by Dixon technique with 2-echoes and 6-echoes at 13 and 17 min after clinically recommended dose of Gx (0.025 mmol/kg) enhancement by which we believed it to be on behalf of the time interval from 10 to 20 min to a great degree. The aims of our study were to clarify to what extent different echoes and flip angles of Dixon in the different time points after Gx enhancement affect the accuracy and reliability of PDFF measurement.

## Methods

This study was approved by our institutional animal care and use committee and was performed in accordance with the NIH guidelines.

### Animal model and experimental design

Forty-two healthy male New Zealand White Rabbits (6–8 months age, bodyweight 2.2–2.5 kg) were from the experimental animal center of experimental animal center of Air Force Medical University. After entering the laboratory, the animals were raised for a week to adapt to the environment, with single cage feeding, free diet and water intake. The conditions in the laboratory were set as follows: temperature of 16–26 ℃, relative humidity of 40%–70%, ventilation rate of 8–10 times/hour, and day/night alternation of 12/12.

Rabbits were randomly divided into control group (*n* = 10) and high-fat diet group (*n* = 32). MR imaging was performed 3 weeks after the control group was fed with a standard diet. High-fat diet group was fed with high-fat diet (88% standard diet + 10% lard + 2% cholesterol) [19]. After such diet, MR imaging was performed at 4 weeks, 8 weeks, 12 weeks, and 16 weeks (*n* = 8, each) respectively.

### Imaging protocols

All examinations were performed on 3.0T MR with a 15-channel knee coil. The animals were fixed on rabbit holder in a supine position for scanning after being anesthetized by intramuscular injection of 2% xylazine hydrochloride (0.3 ml/kg) and ketamine hydrochloride (30 mg/kg).

Taking axial T1-VIBE as the image, imaging was performed before Gx (Gd-EOB- DTPA, Primovist^®^, Bayer Healthcare, Berlin, Germany) enhancement (Pre-Gx), at the 13th (Post-Gx13) and 17th (Post-Gx17) min after enhancement with 2E-VIBE Dixon and 6E-VIBE Dixon (2E/6E-VIBE Dixon) to determine the hepatic PDFFs. In a bolus injection for the enhanced scan, the Gx (0.025 mmol/kg) contrast medium was administered to rabbits in the ear vein, followed by an injection of 6ml saline (1.5 ml/s) through a high-pressure syringe, after which the scan started. The MRI parameters are shown in Table 1.

**Table 1:** MRI pulse sequences and parameters

Parameters	2E-VIBE Dixon	6E-VIBE Dixon	T1-VIBE
TR (ms)	8	13	3.06
TE (ms)	2.46;3.69	1.07;2.96;4.85;6.76;8.62;10.53	1.07
FA (°)	10	5	9
Matrix	192 × 192	192 × 192	192 × 192
No. of signals acquired	2	2	1
FOV (mm)	210 × 210	210 × 210	210 × 210
No. of sections	32	32	32
Thickness (mm)	3	3	3
Section gap	20%	20%	20%
Scan time (s)	36	47	8

*VIBE* volumetric interpolated breath-hold examination, *TR* repetition time, *TE* echo time, *FA* flip angle, *FOV* field of view

### Image analysis

Two radiologists performed image analysis (X.W and YD.H) blinded to the histopathologic results in consensus with more than 5 years of experience in abdominal MR imaging on postprocessing workstation (Syngo.via, Siemens Medical Solutions). The images of Pre-Gx and Post-Gx, both from the 2E- and 6E-VIBE Dixon sequences, were processed using the software provided by the manufacturer to create different maps automatically. For 2E-VIBE Dixon scanning, four maps were generated, including water, fat, in-phase, and out-of-phase maps. For 6E-VIBE Dixon scanning, six maps were generated, including water, fat, water fraction, fat fraction, *r*^2^*, and Goodness of Fit maps. Three circular regions of interest (ROI) were selected in the left lateral, medium, and right lateral liver lobes on the images above. ROIs were initially placed on the Pre-Gx maps, and then copied to the corresponding locations on the Post-Gx maps. PDFFs, R2* (1/T2*), signal intensities of water and fat, were measured to achieve their mean values for subsequent analysis. The main blood vessels and obvious artifacts were avoided for each ROI.

### Histopathological examination

The animals were euthanized by intravenous injection of sodium pentobarbital within 3 h after MR examination. Three liver tissue samples were excised from the left lateral, medium, and right lateral liver lobes of each rabbit. These samples were made into paraffin sections according to the routine procedure of pathological examination. The liver tissue was evaluated by pathologists (NN.L and XM.L) blinded to the diet and the imaging result of each rabbit in consensus with more than 5 years of liver disease research. The liver steatosis degree was observed by HE (Hematoxylin–eosin) staining: Grade 0 (normal): less than 5% of hepatocytes developed steatosis; grade 1 (mild hepatic adipose infiltration): 5%–33% of hepatocytes developed steatosis; grade 2 (moderate hepatic adipose infiltration): 34%–66% of hepatocytes developed steatosis; grade 3 (severe hepatic adipose infiltration): more than 66% of hepatocytes developed steatosis [20]. Using Image Pro-Plus Software 6.0 (Media Cybernetics, CA, United States), 5 representative microscopic fields were selected to calculate the vacuole percentage (VP) of fat vacuole area to total area in the field, average value was taken to evaluate the fat infiltration. Considering that 34.5% of patients with NAFLD were complicated with liver iron deposition [21], from which T2* value and afterwards PDFF being affected, our pathologists also observed whether iron particles existed in the liver and its degree thereof under Prussian blue staining according to Deugnier’s and Turlin’s histological scoring system [22].

### Statistical analysis

Mean ± SD was used for all continuous data. Box plots were created to compare Pre-/Post-Gx PDFFs acquired by 2E-/6E-VIBE Dixon. The distributions of PDFFs acquired by 2E-/6E-VIBE Dixon and VPs were tested using the Kolmogorov–Smirnov test in order to select the proper method of correlation analysis. Correlation coefficient was used to compare the relationships between Pre-/Post-Gx PDFFs measured by 2E-/6E-VIBE Dixon and VPs in histopathology. Repetitive measure analysis of variance was used to evaluate the differences of PDFFs on Pre-Gx and Post-Gx 2E-/6E-VIBE Dixon, and signal intensities of the hepatic water and fat on Pre-Gx and Post-Gx 2E-VIBE Dxion. Bland–Altman analysis was used to evaluate the consistency of PDFFs obtained by 2E-/6E-VIBE Dixon. Limits of agreement (95%) were defined as the mean difference ± 1.96 × the standard deviation. Statistical results were significant at *P* < 0.05. All statistical analyses were performed by using SPSS 23.0 software (SPSS, Chicago, Ill).

## Results

### Animal model and pathological grouping

All 10 control group animals went through the experiment alive. In the high-fat diet group, 9 animals died, and 23 animals went through the experiment alive. MASLD model (7 mild hepatic adipose infiltration, 10 moderate hepatic adipose infiltration, 5 severe hepatic adipose infiltration) was successfully established on 22 animals, and 1 animal showed normal pathology and was classified into the control group.

**Fig. 1:**
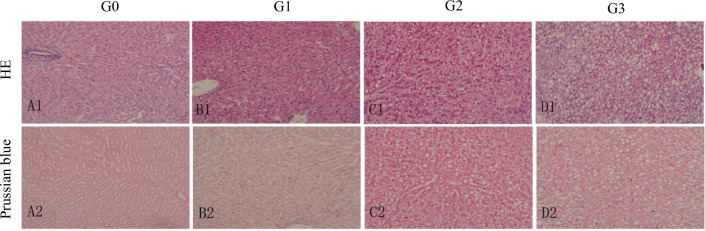
Representative histopathologic slices of rabbit liver steatosis stained by haematoxylin–eosin (HE, ×200, **A1–D1**) and Prussian blue (**A2–D2**, ×200). There were no fat vacuoles and iron deposition in normal liver tissue (G0, **A1**, **A2**); Fat vacuoles gradually increased in the adipose liver with G1 (**B1**, **B2**) and G2 (**C1**, **C2**) stages, and no iron deposition was found; Fat vacuoles in the cytoplasm of hepatocytes had a significant increase and aggregation in the adipose liver with G3 (**D1**, **D2**) stages, and the iron particles colored with blue staining were scattered. *G* grade

HE staining showed that the structure of the normal lobuli hepatis was clear, and there was no obvious lipid droplet or fat vacuole in the cytoplasm of hepatocytes. In MASLD rabbits, lipid droplets and fat vacuoles in hepatocytes were scattered and partially fused (Fig. 1A1–D1). Prussian blue staining showed no obvious iron deposition in the liver of all animals (Fig. 1A2–D2).

### PDFF changes at different measurement time points before and after Gx enhancement

**Fig. 2:**
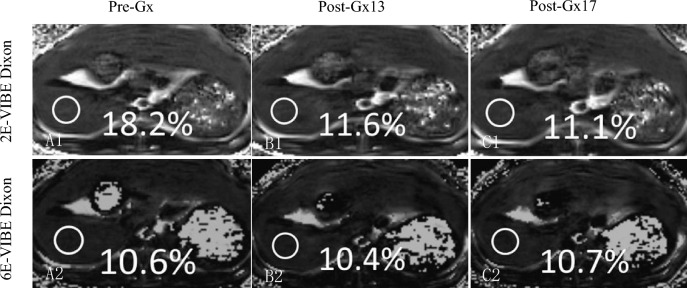
Example of proton density fat fraction (PDFF) maps acquired with 2E- and 6E-VIBE Dixon, before and after administration of Gadoxetate disodium in a rabbit with hepatic steatosis. On 2E-VIBE Dixon, PDFF was apparently overestimated before Gx enhancement (**A1**), and, decreased after enhancement (**B1**, **C1**) and tended to be consistent with that of 6E-VIBE Dixon. On 6E-VIBE Dixon, however, PDFFs were stable without significant change from Pre-Gx to Post-Gx imaging (**A2**–**C2**). *Pre-Gx* before Gx administration, *Post-Gx13* 13min. after Gx administration, *Post-Gx17* 17 min after Gx administration

**Fig. 3:**
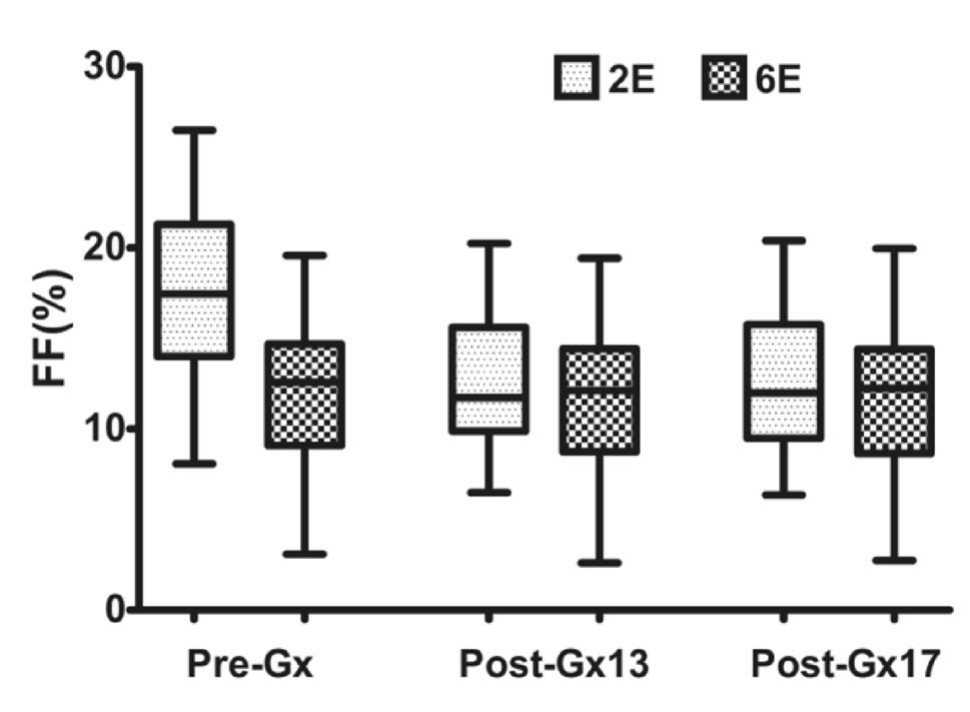
Box plots showed the accuracy of PDFFs quantified by 2E-VIBE Dixon and 6E-VIBE Dixon, in which the border of the box indicated the upper and lower quartiles, and the center line inside the box indicated the median values. *FF* fat fraction, *2E* 2E-VIBE Dixon, *6E* 6E-VIBE Dixon, *Pre-Gx* before Gx administration, *Post-Gx 13* 13min. after Gx administration, *Post-Gx17* 17min. after Gx administration

The PDFFs measured by 2E-VIBE Dixon were higher than their corresponding 6E-VIBE Dixon’s, whether before or after Gx enhancement (Fig. 2). Variance analysis and box analysis were performed on PDFFs measured by 2E/6E-VIBE Dixon at Pre-Gx, Post-Gx13,and Post-Gx17in MASLD rabbits, showing that 2E-VIBE Dixon PDFFs reduced significantly after enhancement (*P* < 0.001), but there was no significant difference at the 13th and 17th minute after enhancement (*P* = 0.123). 6E-VIBE Dixon PDFFs changed little (*P* = 0.322) (Fig. 3).

### Signal intensity analysis of water and fat before and after enhancement on 2E-VIBE Dixon

On the water image and fat image of 2E-VIBE Dixon, the signal intensities of water and fat were further analyzed respectively before and after Gx enhancement. The results (Table 2) showed that Gx enhancement had significant influence on the water signal intensity. Postcontrast signal intensity was markedly higher than precontrast signal intensity (*P* < 0.001), but there was no significant difference at the 13th and 17th minute after enhancement (*P* = 0.073). However, Gx enhancement had no significant influence on fat signal intensity (*P* = 0.754).

**Table 2:** Signal intensities before and after Gx enhancement on 2E-VIBE Dixon

VIBE Dixon (*n* = 22)	$\overline{X }\pm S (\%)$	*F*	*P*
Pre-Gx-W	206.92 ± 18.22	87.130	< 0.001
Post-Gx13-W	307.73 ± 31.32		
Post-Gx17-W	303.50 ± 31.08		
Pre-Gx-F	44.60 ± 15.41	0.263	0.754
Post-Gx13-F	44.68 ± 15.13		
Post-Gx17-F	44.67 ± 15.21		

*Pre-Gx* before Gx administration, *Post-Gx13* 13min. after Gx administration, *Post-Gx17* 17min. after Gx administration, *W* water, *F* fat

### Correlations between PDFFs and VPs

**Fig. 4:**
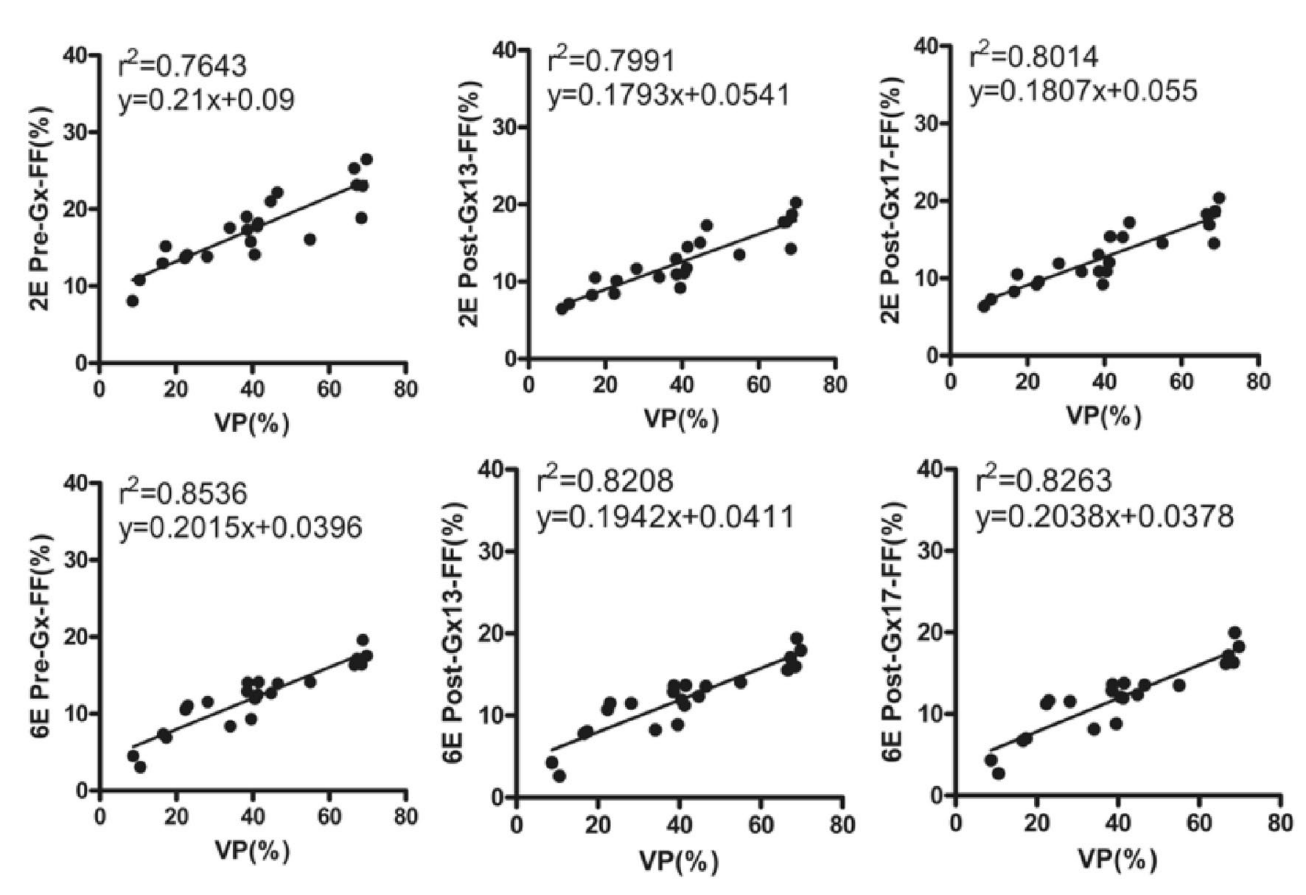
Correlations between the PDFFs and VPs in the group of MASLD rabbits. Excellent correlations were found before and after Gx administration assessed by 6E-VIBE Dixon, with the best one of *r*^2^ = 0.8536 on Pre-Gx 6E-VIBE Dixon. However, relatively lower correlation coefficients were found before and after Gx administration assessed by 2E-VIBE Dixon, with the lowest one of *r*^2^ = 0.7643 on Pre-Gx 2E-VIBE Dixon. *VP* vacuole percentage, *FF* fat fraction, *2E* 2E-VIBE Dixon, *6E* 6E-VIBE Dixon, *Pre-Gx* before Gx administration, *Post-Gx 13* 13min. after Gx administration, *Post-Gx 17* 17min. after Gx administration

The PDFF results and VP results summarized in Table 3. In MASLD rabbits (*n* = 22), PDFFs obtained by 2E-/6E-VIBE Dixon (before and after Gx enhancement) and VPs in histopathology had a positive correlation (*P* < 0.001), wherein *r*^2^ was 0.7643–0.8014 and 0.8208–0.8536 respectively (Fig. 4). The highest correlation between the PDFF and VP was found in Pre-Gx (*r*^2^ = 0.8536) on 6E-VIBE Dixon and the lowest one in Pre-Gx (*r*^2^ = 0.7643) on 2E-VIBE Dixon. Additionally, no significant iron deposition was found on the R2* image of 6E-VIBE Dixon in the animals of control group and high-fat diet group.

**Table 3:** The hepatic biomarkers of PDFFs on MRI and VPs on histopathology (mean ± SD)

G	*N*	VP (%)	Pre-Gx		Post-Gx13		Post-Gx17	
			2E	6E	2E	6E	2E	6E
0	11	2.61 ± 1.16	5.13 ± 1.42	2.10 ± 0.73	2.89 ± 0.87	2.07 ± 0.74	3.13 ± 0.92	2.10 ± 0.67
1	7	18.09 ± 6.45	12.66 ± 2.43	7.85 ± 3.33	8.94 ± 1.89	8.06 ± 3.53	9.01 ± 1.90	7.91 ± 3.66
2	10	42.05 ± 5.40	17.91 ± 2.40	12.40 ± 2.03	12.68 ± 2.44	12.05 ± 2.03	12.94 ± 2.58	12.08 ± 2.01
3	5	68.16 ± 1.14	23.37 ± 2.91	17.41 ± 1.18	17.74 ± 2.20	17.19 ± 1.40	17.76 ± 2.20	17.58 ± 1.40

*G* grade, *N* number, *VP* vacuole percentage, *2E* 2E-VIBE Dixon, *6E* 6E-VIBE Dixon, *Pre-Gx* before Gx administration, *Post-Gx13* 13min. after Gx administration, *Post-Gx17* 17min. after Gx administration

### Bland–Altman analysis

**Fig. 5:**
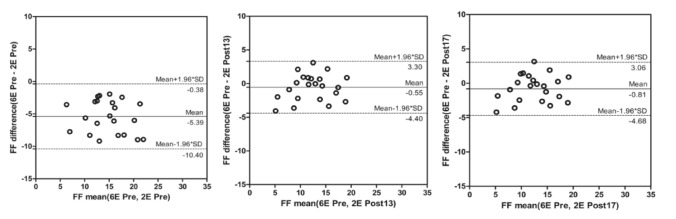
Bland–Altman analysis of PDFFs for Pre-Gx and Post-Gx 2E-VIBE Dixon versus 6E-VIBE Dixon. The middle solid line indicated the mean difference, and the dotted line denoted 95% limits of agreement (mean difference ± 1.96 × the standard deviation). Compared with that on Pre-Gx 2E-VIBE Dixon, PDFFs of Post-Gx13 and Post-Gx17 had better consistency with that on Pre-Gx 6E-VIBE Dixon. *FF* fat fraction, *2E* 2E-VIBE Dixon, *6E* 6E-VIBE Dixon, *Pre-Gx* before Gx administration, *Post-Gx 13* 13min. after Gx administration, *Post-Gx 17* 17min. after Gx administration

The Bland–Altman analysis was used to compare the PDFFs by 2E-VIBE Dixon at Pre-Gx, Post-Gx13 and Post-Gx17 with the PDFF by 6E-VIBE Dixon before enhancement (Fig. 5).The mean difference was (− 5.39 ± 2.56)% (95% CI − 0.38 to − 10.40%), (− 0.55 ± 1.97)% (95% CI 3.30 to − 4.40%), and (− 0.81 ± 1.97)% (95% CI 3.06 to − 4.68%) respectively. After enhancement, PDFFs on 2E-VIBE Dixon had the better consistency compared with that on 6E-VIBE Dixon before enhancement. All measurements showed the same except that on 2E-VIBE Dixon before enhancement which had higher PDFF values.

## Discussion

Our results showed that reliable models of MASLD with varying degrees of severity could be produced by New Zealand White rabbits. Marked liver deposition of iron was not found in all these rabbits. PDFFs changed little (*P* = 0.322) on 6E-VIBE Dixon before and after Gx enhancement in MASLD rabbits, in which the best correlation with the histopathologic result was found in precontrast (*r*^2^ = 0.8536). However, PDFFs changed significantly (*P* < 0.001) on 2E-VIBE Dixon before and after Gx enhancement, in which the lowest correlation with the histopathologic result being found in precontrast (*r*^2^ = 0.7643), and the higher correlations closed to that of 6E-VIBE Dixon being found in postcontrast (*r*^2^ = 0.7991/0.8014). On the water map and fat map of 2E-VIBE Dixon, the signal intensity of water increased (*P* < 0.001) significantly after Gx enhancement, while the signal intensity of fat did not (*P* = 0.754).

The PDFFs of liver measured by 2E-VIBE Dixon (FA = 10°) after Gx enhancement were significantly lower than that before Gx enhancement (*P* < 0.001). This change may be due to the dipole–dipole interaction between Gx and water molecules, which significantly shortens the T1 value of water molecules but has no significant effect on the T1 value of fat [14, 23, 24]. Park et al. [25] found that there was no significant change in mean fat T1 value of liver before and after Gx enhancement (*P* = 0.389), while water T1 value decreased from 969 ± 155 to 361 ± 105 ms (*P* < 0.001), similar to fat T1 value (*P* = 0.326). On the water images and fat images under 2E-VIBE Dixon, we found that the signal intensity of water increased significantly after enhancement (*P* < 0.001), but the signal intensity of fat didn’t change significantly (*P* > 0.001), indicating that Gx effectively minimized T1 bias by promoting the recovery of longitudinal magnetization of water. There was no statistical difference (*P* = 0.322) in PDFFs quantified with 6E-VIBE Dixon (FA = 5°) before and after enhancement, resulting in a good correlation with pathological results (*r*^2^ = 0.8208–0.8536), which may be related to the reduction of T1 value because of the small flip angle [17, 18, 25, 26].

After Gx enhancement, there was a long window period between the hepatic conventional three-phase scanning and the specific hepatobiliary phase scanning 20 min later. Effective use of this period can improve the efficiency of examination. Research is rare on whether hepatic PDFF is affected by the contrast medium above and the extent to which it is affected. Hernando et al. [18] compared the hepatic PDFF around 20min after Gx administration (0.05 mmol/kg) with that under no Gx administration using water-fat separation technique (FA = 5°), finding that PDFFs showed no significant difference (*P* = 0.320). The reason of the phenomenon was due to the small flip angle by which the effects of T1 related bias could be avoided [18]. In our study, we adopted clinically recommended dose of Gx (0.025 mmol/kg) and set the observation time points at the 13th and 17th minute after enhancement. Our results showed that PDFFs maintained a good consistency and reliability (*P* > 0.001) at this two moments, and had good correlations with the histopathologic findings, whether under 2E-VIBE Dixon (FA = 10°) or 6E-VIBE Dixon (FA = 5°). We think that it was due to the minimization effect to the T1 related PDFF bias of Gx under the circumstance of relatively large flip angle (FA ≥ 10°). And this suppression of T1 effects could be stablely and accurately sustained from Post-Gx13 to Post-Gx 17. Combined with the research of Hernando et al. [18] hepatic PDFFs might be reliably achieved at the window period of 13 to 20 min in the hepatobiliary phase after Gx enhancement.

In our study, only scattered iron particles were occasionally found in the animal livers. Hepatic PDFFs were not underestimated both on the 2E-/6E-VIBE Dixon. Our study also showed no significant iron deposition on the R2* map of 6E-VIBE Dixon, similar to that of the histopathologic results. Clinically, however, hepatic iron deposition could be occurred in about one third of the patients with MASLD [21, 27]. Although the association of degree of hepatic iron deposition with the severity of MASLD remains controversial, mild to moderate hepatic iron accumulation, especially the former, is the typical histological finding [21, 27]. Iron can shorten T2* value for the liver tissue, and the more the T2* signal is lost, the smaller the hepatic PDFF value is [10–12, 28]. Some researchers found that T2* correction can be corrected by multi-echo technology with more than three echoes, but it cannot be done by dual echo Dixon [10, 12, 17]. In the study reported by Kukuk et al. [10] the hepatic PDFF that was underestimated with 2E-mDixon because of the presence of iron, had a good correlation with MRS (*r* = 0.984) and histopathologic results (*r* = 0.967) on 6E-mDixon.Therefore,the multi-echo Dixon should be used to quantify the hepatic PDFF to correct the influence of possibly existed iron deposition in the liver [12, 17, 21].

This study has some limitations. Firstly, the hepatic PDFFs at the 13th and 17th minute after Gx enhancement were observed, but PDFF changes at other time points in the hepatobiliary specific phase were not covered. Secondly, different functional states of hepatocytes may affect PDFF after Gx enhancement [29, 30], which was not discussed herein. Thirdly, in this study, we found that the correlations between PDFFs measured with 6E-VIBE Dixon (FA = 5°) after Gx enhancement and VPs under histopathology decreased slightly, which needs to be further studied. And the last one, the relatively small sample size of animal models perhaps might not reflect the complicated presentations of the liver steatosis. Much more research on animal models and even human beings should be further studied in the future.

## Conclusions

Our research showed that the hepatic PDFFs quantified with 6E-VIBE Dixon could be accurately obtained both before or after Gx enhancement. On 2E-VIBE Dixon, however, PDFFs were significantly influenced by Gx administration. More accurate hepatic PDFFs could be achieved at 13 and 17 min after Gx enhancement compared with that before enhancement. Therefore, the window period from 13 to 17th minute of the hepatobiliary specific phase can be efficiently utilized to quantify the hepatic PDFFs by both 2E-VIBE Dixon and 6E-VIBE Dixon.

## Data Availability

The datasets generated or analyzed during the study are available from the corresponding author on reasonable request.

## Abbreviations

MASLD: Metabolic dysfunction-associated steatotic disease
PDFF: Proton density fat fraction
Gx: Gadoxetate disodium
VP: Vacuole percentage
Pre-Gx: Before the administration of Gadoxetate disodium
Post-Gx13: 13 Min after the administration of Gadoxetate disodium
Post-Gx17: 17 Min after the administration of Gadoxetate disodium
VIBE: Volumetric interpolated breath-hold examination
2E: 2 Echoes
6E: 6 Echoes

## References

[1] Rinella ME, Lazarus JV, Ratziu V et al (2023) A multisociety Delphi consensus statement on new fatty liver disease nomenclature. Hepatology 78: 1966-1986. 10.1097/HEP.0000000000000520. 37363821 10.1097/HEP.0000000000000520 PMC10653297

[2] Younossi ZM, Koenig AB, Abdelatif D et al (2016) Global epidemiology of nonalcoholic fatty liver disease-Meta-analytic assessment of prevalence, incidence, and outcomes. Hepatology 64(1):73-84. 10.1002/hep.28431. 26707365 10.1002/hep.28431

[3] Marcellin P, Kutala BK (2018) Liver diseases: A major, neglected global public health problem requiring urgent actions and large-scale screening. Liver Int 38 Suppl 1:2-6. 10.1111/liv.13682. 29427496 10.1111/liv.13682

[4] Younossi ZM (2019) Non-alcoholic fatty liver disease - A global public health perspective. J Hepatol 70:531-544. 10.1016/j.jhep.2018.10.033. 30414863 10.1016/j.jhep.2018.10.033

[5] Longo R, Pollesello P, Ricci Cet al (1995) Proton MR spectroscopy in quantitative in vivo determination of fat content in human liver steatosis. J Magn Reson Imaging 5:281-285. 10.1002/jmri.1880050311. 7633104 10.1002/jmri.1880050311

[6] Reeder SB, Cruite I, Hamilton G et al (2011) Quantitative assessment of liver fat with magnetic resonance imaging and spectroscopy. J Magn Reson Imaging 34:729-749. 10.1002/jmri.22775. 22025886 10.1002/jmri.22775 PMC3177109

[7] Wáng YXJ, Wang X, Wu Pet al (2019) Topics on quantitativelivermagnetic resonance imaging. Quant Imaging Med Surg 9:1840-1890. 10.21037/qims.2019.09.18. 31867237 10.21037/qims.2019.09.18 PMC6902134

[8] Idilman IS, Keskin O, Celik A et al (2016) A comparison of liver fat content as determined by magnetic resonance imaging-proton density fat fraction and MRS versus liver histology in non-alcoholic fatty liver disease. Acta Radiol 57:271-278. 10.1177/0284185115580488. 25855666 10.1177/0284185115580488

[9] Dixon WT (1984) Simple proton spectroscopic imaging. Radiology 153: 189-194. 10.1148/radiology.153.1.6089263. 6089263 10.1148/radiology.153.1.6089263

[10] Kukuk GM, Hittatiya K, Sprinkart AM et al (2015) Comparison between modified dixon MRI techniques, MR spectroscopic relaxometry, and different histologic quantification methods in the assessment of hepatic steatosis. Eur Radiol 25:2869-2879. 10.1007/s00330-015-3703-6. 25903702 10.1007/s00330-015-3703-6

[11] Liu CY, McKenzie CA, Yu H et al (2007) Fat quantification with ideal gradient echo imaging: correction of bias from T(1) and noise. Magn Reson Med 58:354-364. 10.1002/mrm.21301. 17654578 10.1002/mrm.21301

[12] Yu H, McKenzie CA, Shimakawa A et al (2007) Multiecho reconstruction for simultaneous water-fat decomposition and T2* estimation. J Magn Reson Imaging 26:1153-1161. 10.1002/jmri.21090. 17896369 10.1002/jmri.21090

[13] Yokoo T, Serai SD, Pirasteh A et al (2018) Linearity, bias, and precision of hepatic proton density fat fraction measurements by using MR imaging: a meta-analysis. Radiology 286: 486-498. 10.1148/radiol.2017170550. 28892458 10.1148/radiol.2017170550 PMC5813433

[14] Hamm B, Staks T, Mühler A et al (1995) Phase I clinical evaluation of Gd-EOB-DTPA as a hepatobiliary MR contrast agent: safety, pharmacokinetics, and MR imaging. Radiology 195:785-792. 10.1148/radiology.195.3.7754011. 7754011 10.1148/radiology.195.3.7754011

[15] Reimer P, Rummeny EJ, Shamsi K et al (1996) Phase II clinical evaluation of Gd-EOB-DTPA: dose, safety aspects, and pulse sequence. Radiology 199:177-183. 10.1148/radiology.199.1.8633143. 8633143 10.1148/radiology.199.1.8633143

[16] Ringe KI, Husarik DB, Gupta RT et al (2011) Hepatobiliary transit times of gadoxetate disodium (Primovist®) for protocol optimization of comprehensive MR imaging of the biliary system--what is normal? Eur J Radiol 79(2):201-205. 10.1016/j.ejrad.2010.03.008. 20347540 10.1016/j.ejrad.2010.03.008

[17] Hayashi T, Fukuzawa K, Kondo H et al (2017) Influence of Gd-EOB-DTPA on proton density fat fraction using the six-echoDixon method in 3 Tesla magnetic resonance imaging. Radiol Phys Technol 10:483-488. 10.1007/s12194-017-0420-7. 28895045 10.1007/s12194-017-0420-7

[18] Hernando D, Wells SA, Vigen KK et al (2015)Effect of hepatocyte-specific gadolinium-based contrast agents on hepatic fat-fraction and R2(⁎). Magn Reson Imaging 33:43-50. 10.1016/j.mri.2014.10.001. 25305414 10.1016/j.mri.2014.10.001 PMC4268245

[19] Fu JF, Fang YL, Liang L et al (2009) A rabbit model of pediatric nonalcoholic steatohepatitis: the role of adiponectin. World J Gastroenterol 15:912-918. 10.3748/wjg.15.912. 19248189 10.3748/wjg.15.912 PMC2653407

[20] Brunt EM, Janney CG, Di Bisceglie AM et al (1999) Nonalcoholic steatohepatitis: a proposal for grading and staging the histological lesions. Am J Gastroenterol 94: 2467-2474. 10.1111/j.1572-0241.1999.01377.x. 10484010 10.1111/j.1572-0241.1999.01377.x

[21] Nelson JE, Klintworth H, Kowdley KV (2012) Iron metabolism in nonalcoholic fatty liver disease. Curr Gastroenterol Rep 14:8-16. 10.1007/s11894-011-0234-4. 22124850 10.1007/s11894-011-0234-4

[22] Deugnier Y, Turlin B (2007) Pathology of hepatic iron overload. World J Gastroenterol 3:4755-4760. 10.3748/wjg.v13.i35.4755. 10.3748/wjg.v13.i35.4755 PMC4611197 17729397

[23] Haimerl M, Probst U, Poelsterl S et al (2018) Evaluation of two-point Dixon water-fat separation for liver specific contrast-enhanced assessment of liver maximum capacity. Sci Rep 8:13863. 10.1038/s41598-018- 32207-6. 30218001 10.1038/s41598-018-32207-6 PMC6138716

[24] Caravan P, Ellison JJ, McMurry TJ et al (1999) Gadolinium(III) Chelates as MRI contrast agents: structure, dynamics, and applications. Chem Rev 99:2293-2352. 10.1021/cr980440x. 11749483 10.1021/cr980440x

[25] Park CC, Hamilton G, Desai A et al (2017) Effect of intravenous gadoxetate disodium and flip angle on hepatic proton density fat fraction estimation with six-echo, gradient-recalled-echo, magnitude-based MR imaging at 3T. Abdom Radiol 42:1189-1198. 10.1007/s00261-016-0992-4. 10.1007/s00261-016-0992-4 28028556

[26] Wang X, Colgan TJ, Hinshaw LA et al (2020) T1-corrected quantitative chemical shift-encoded MRI. Magn Reson Med 83:2051-2063. 10.1002/mrm.28062. 31724776 10.1002/mrm.28062 PMC7047527

[27] Younossi ZM, Gramlich T, Bacon BR et al (1999) Hepatic iron andnonalcoholic fatty liver disease. Hepatology 30:847-850. 10.1002/hep.510300407. 10498632 10.1002/hep.510300407

[28] Henninger B, Kremser C, Rauch S et al (2013) Evaluation of liver fat in the presence of iron with MRI using T2* correction: a clinical approach. Eur Radiol 23:1643-1649. 10.1007/s00330-012-2745-2. 23334458 10.1007/s00330-012-2745-2

[29] Haimerl M, Verloh N, Zeman F et al (2017) Gd-EOB-DTPA-enhanced MRI for evaluation of liver function: comparison between signal-intensity-based indices and T1 relaxometry. Sci Rep 7:43347. 10.1038/ srep43347. 28266528 10.1038/srep43347 PMC5339723

[30] Yoo H, Lee JM, Yoon JH et al (2017) T2* Mapping from Multi-Echo Dixon sequence on Gadoxetic acid-enhanced magnetic resonance imaging for the hepatic fat quantification: can it be used for hepatic function assessment? Korean J Radiol 18:682‐690. 10.3348/kjr.2017.18.4.682. 28670163 10.3348/kjr.2017.18.4.682 PMC5447644

